# The effect of depression on quality of life in infertile couples: an actor-partner interdependence model approach

**DOI:** 10.1186/s12955-018-0904-0

**Published:** 2018-04-24

**Authors:** Saman Maroufizadeh, Mostafa Hosseini, Abbas Rahimi Foroushani, Reza Omani-Samani, Payam Amini

**Affiliations:** 1 Department of Epidemiology and Biostatistics, School of Public Health, Tehran University of Medical Sciences, Tehran, Iran; 2 Department of Epidemiology and Reproductive Health, Reproductive Epidemiology Research Center, Royan Institute for Reproductive Biomedicine, ACECR, Tehran, Iran

**Keywords:** Quality of life, Depression, Infertility, Actor–partner interdependence model

## Abstract

**Background:**

Infertility can cause psychological distress and has a negative impact on quality of life (QoL). There have been no studies investigating the effect of depression on QoL in infertile couples at the dyadic level. This study aimed to investigate the effects of actors’ and partners’ depression on QoL in male-female dyads experiencing infertility using an innovative dyadic analysis approach, the Actor–Partner Interdependence Model (APIM).

**Methods:**

We conducted this cross-sectional study on 180 infertile couples in Tehran, Iran, during August-September 2017. Quality of life and depression were assessed using Fertility Quality of Life and Patient Health Questionnaire-9, respectively. Dyadic data were analyzed by the APIM approach. In this method, actor effect is the impact of a person’s depression on his/her own QoL. Partner effect is the impact of a person’s depression on his/her partner’s QoL.

**Results:**

Results from APIM revealed that both males and females’ depression exuded an actor effect on their own QoL (β = − 0.589, *p* < 0.001; β = − 0.588, *p* < 0.001, respectively). Furthermore, males’ depression exuded a significant partner effect on their wives’ QoL (β = − 0.128, *p* = 0.030). Although the partner effect of females’ depression on males’ QoL was not statistically significant (β = − 0.108, *P* = 0.070), males whose wives had higher depression were more to indicate their own QoL was poorer. Based on equality constraint test, both actor and partner effects of depression on QoL were similar between males and females.

**Conclusions:**

The findings suggest that QoL in infertile patients was influenced by not only their own depression but also their spouses’ depression; therefore, interventions to improve QoL should include both males and females.

## Background

Infertility is defined by the World Health Organization as “the failure to achieve a clinical pregnancy after 12 months or more of regular unprotected sexual intercourse” [1]. It is a public health problem affecting 9% of reproductive-aged couples throughout the world [2]. A growing body of research suggests that both infertility and its treatment represent a negative psychological burden to affected couples and this can has a considerable impact on their quality of life (QoL), life satisfaction and well-being [3, 4]. One of the most often-cited repercussions of infertility is depressive disorder, and increasing evidence indicates that this disorder is associated with poor QoL in people with infertility [5–7].

Many of the phenomena studied by researchers in social and behavioral science are dyadic in nature (e.g., research on male-female dyads and parent-child dyads). The observations arising from such designs are not independent, but interdependent; however, in this case independence refers to independence from dyad to dyad [8, 9]. Statistically, conventional parametric statistics developed for independent observations are not appropriate for non-independent observations. Instead, the non-independence due to the dyadic nature of data must be taken into account when relationships are investigated. One situation, in which “non-independence” is relevant, is that a characteristic or behavior of one person affects his or her partner’s outcomes; therefore, a model that takes non-independence into account is needed for an accurate analysis [9]. The Actor–Partner Independence Model (APIM), an innovative dyadic analysis approach, simultaneously estimates the effects of one’s own characteristics and one’s partner’s characteristics on an outcome variable [9]. The APIM approach uses the dyad, and not the individual, as the sampling unit and provides separate but simultaneous estimates of actor and partner effects [9]. The actor effect assesses the degree to which one’s outcome is influenced by one’s own characteristics, whereas the partner effect assesses the degree to which a person’s outcome is influenced by characteristics of the partner.

Most studies evaluating the relationships between depression and QoL in infertile couples use the individual as the unit of analysis [10, 11]. Although valuable, these studies provide no information on the impact that partner depression has on individual QoL. In addition, since infertility is a shared couple problem, examining the impact of partner depression is especially relevant [12]. In other words, the male’s/female’s depression does not only influence his/her own QoL, but also his/her partner’s QoL. Thus, the current study aimed to: (a) evaluate whether there were differences in the levels of depression and QoL between male and female dyads experiencing infertility; (b) use the APIM approach to elucidate and differentiate actor effects and partner effects of depression on QoL. In this study, we examine the following research hypotheses: (1) There is a significant difference between males and females’ depression; (2) There is a significant difference between males and females’ QoL; (3) One’s level of depression is associated with his/her own level of QoL (actor effects); (4) One’s level of depression is associated with his/her spouse’s level of QoL (partner effects); (5) There is a significant difference between male and female actor effects of depression on QoL; (6) There is a significant difference between male and female partner effects of depression on QoL; (7) There is a significant difference between actor effect and partner effect separately for both males and females.

## Methods

### Participants and study design

We conducted this cross-sectional study on infertile couples who were referred to the Infertility Treatment Center of Royan Institute, a referral center for infertility treatment in Tehran, Iran [13]. The data were collected using a convenience sampling method between August to September 2017. The eligibility criteria in this study were as follows: (a) age > 18 years; (b) experiencing infertility; (c) willingness to take part in the study; (d) ability to read, write, and comprehend Persian. Infertile couples were asked to filled out the instruments without discussing their answers with each other. In total, 180 infertile couples agreed to take part in the study and filled out the instruments completely (response rate: 81.8%).

### Ethical consideration

This study was approved by the Ethics Committee of Tehran University of Medical Sciences, Tehran, Iran. The couples were informed of the aim of the study and of their right to refrain from participation and were assured of confidentiality and anonymity. Agreement to participate and a signed consent form were obtained from all infertile couples before data collection.

### Instruments

#### Fertility Quality of Life (FertiQoL)

FertiQoL is a disease-specific self-administered tool that assesses for QoL in people experiencing fertility problems [14]. This scale consists of two modules: The Core FertiQoL and the Treatment FertiQoL. The Core FertiQoL module yields four subscales (Emotional, Mind-Body, Relational, and Social). Each subscale consists of 6 items and respondents answer each item using a 5-point Likert scale ranging from 0 to 4. The optional Treatment module, which assesses accessibility/quality of treatment and burden/tolerability of fertility treatment, was not used in the present study. Raw total scores as well as its subscales scores were scaled to range from 0 to 100, with higher score representing better QoL. The Persian version of the FertiQoL has shown adequate reliability and validity [15]. Internal consistency of the FertiQoL was high in the present study (Cronbach’s α = 0.905).

#### Patient Health Questionnaire-9 (PHQ-9)

The PHQ-9 is a 9-item self-administered tool that assesses for depression based on the DSM-IV criteria for major depressive episode [16]. Respondents rate items on a 4-point Likert scale from 0 (not at all) to 3 (nearly every day) over the past two weeks. The total score ranges from 0 to 27, with high scores representing greater depression symptoms. Internal consistency of the PHQ-9 was high in the present study (Cronbach’s α = 0.874).

### Statistical analysis

#### Preliminary analyses

Comparison of demographics characteristics, depression and QoL for males and females were performed via the McNemar test and paired sample t test. Pearson’s correlation coefficient was calculated to investigate the bivariate relationship among the study variables.

#### The Actor–Partner Interdependence Model (APIM)

**Fig. 1:**
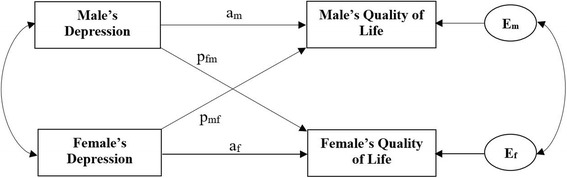
Actor–Partner Interdependence Model of depression and quality of life in infertile couples. **a**_**m**_: actor effect of male’s depression on his own quality of life; **a**_**f**_: actor effect of female’s depression on her own quality of life; **p**_**fm**_: partner effect of male’s depression on female’s quality of life; **p**_**mf**_: partner effect of female’s depression on male’s quality of life; **E**_**m**_ and **E**_**f**_: residual errors on quality of life for males and females, respectively

The APIM with distinguishable dyads [9] was applied to examine the impact of males and females’ depression on their own, as well as their spouse’s QoL. Figure 1 depicts the APIM of a male-female dyad in which there are two variables from each in the dyad: depression (independent variable) and QoL (outcome variable). The male’s level of QoL is affected by his own level of depression (actor effect, a_m_) and by female’s depression (partner effect, p_mf_). Similarly, the female’s level of QoL is influenced by her own depression (actor effect, a_f_) and male’s depression (partner effect, p_fm_).

There are also two important correlations in the model. First, the two independent variables might be correlated, shown by the curved line in Fig. 1, which might be due to a compositional effect. Second, the correlation between the error or residual terms (E_m_ and E_f_), which represents the non-independence beyond that explained by the model.

Three different methods can be used to estimate the APIM: pooled regression modeling, multilevel modeling, and structural equation modeling (SEM). As recommended by Kenny et al. [9], SEM with distinguishable dyads is the simplest data analytic method for estimating the APIM. The SEM approach involves estimating the APIM parameters as they appear in the model presented in Fig. 1. According to the dyad-level structure, two linear equations are as follows:

$$ {\mathrm{Y}}_{\mathrm{m}}={\mathrm{a}}_{\mathrm{m}}\ {\mathrm{X}}_{\mathrm{m}}+{\mathrm{p}}_{\mathrm{m}\mathrm{f}}\ {\mathrm{X}}_{\mathrm{f}}+{\mathrm{E}}_{\mathrm{m}}, $$

$$ {\mathrm{Y}}_{\mathrm{f}}={\mathrm{a}}_{\mathrm{f}}\ {\mathrm{X}}_{\mathrm{f}}+{\mathrm{p}}_{\mathrm{f}\mathrm{m}}\ {\mathrm{X}}_{\mathrm{m}}+{\mathrm{E}}_{\mathrm{f}}, $$

where Y_m_ is the male’s QoL, Y_f_ is the female’s QoL, X_m_ is the male’s depression, and X_f_ is the female’s depression. Because the dyad is the unit of analysis, the sample size in this analysis is the number of couples (which is 180 in this study).

A valuable aspect of the SEM approach is that it allows model constraints to be placed and tested in the APIM framework. For example, it can test whether the male’s actor effect is equal to the female’s actor effect (a_m_ = a_f_) and then measuring the degree to which this constraint significantly worsens the model fit [9, 17]. To test for this gender difference, an equality constraint test was used by the examination of the chi-square difference test. If this test is statistically significant, then that indicates that the actor effects for males and females cannot be the same.

To compute a χ^2^ difference test, the difference of the χ^2^ values of the two models (constrained and unconstrained models) in question is taken as well as the difference of the degrees of freedom.

$$ {\upchi^2}_{\mathrm{diff}}={\upchi^2}_{\mathrm{constrained}}-{\upchi^2}_{\mathrm{unconstrained}} $$

$$ {\mathrm{df}}_{\mathrm{diff}}={\mathrm{df}}_{\mathrm{constrained}}-{\mathrm{df}}_{\mathrm{unconstrained}} $$

All statistical tests were two-sided and a *P*-value < 0.05 was considered statistically significant.

#### Statistical software

All preliminary analyses were carried out using IBM SPSS Statistics for Windows, Version 22.0 (IBM Crop., Armonk, NY, USA), and APIM analysis was carried out using Mplus software version 6.12 (Muthén & Muthén, Los Angeles, CA, USA).

## Results

### Characteristics of the male and female dyads

Table 1 presents the demographic and clinical characteristics of the infertile couples. The males, on average, were 3.77 years older than females (t _(179)_ = 11.94, *P* < 0.001), but had a similar education level as females (χ^2^_(1)_ = 0, *P* = 1.000). The mean duration of marriage and infertility were 6.72 ± 3.94 and 4.83 ± 3.61 years, respectively. Infertility was due to a male or female factor in 45.0 and 17.2% of dyads, respectively. In 12.8%, both male and female factors were observed, and 25.0% of couples had unexplained infertility. Majority of the couples had primary infertility (74.4%), and no history of abortion (77.8%) and 47.2% of them had experienced at least one failure in previous assisted reproductive technology treatments.

**Table 1:** Demographic and clinical characteristics of the male and female dyads (*n* = 180 couples)

	Male	Female	Test statistic	*P*
Age (years), mean ± SD	34.31 ± 5.01	30.54 ± 5.39	t_(179)_ = 11.94	< 0.001
Educational level, *n* (%)			χ^2^_(1)_ = 0	1.000
Non-academic	96 (53.3)	95 (52.8)		
Academic	84 (46.7)	85 (47.2)		
Duration of marriage (years), mean ± SD	6.72 ± 3.94			
Duration of infertility (years), mean ± SD	4.83 ± 3.61			
Cause of infertility, *n* (%)				
Male factor	81 (45.0)			
Female factor	31 (17.2)			
Both	23 (12.8)			
Unexplained	45 (25.0)			
Failure of previous treatment, *n* (%)				
No	95 (52.8)			
Yes	85 (47.2)			
History of abortion, *n* (%)				
No	140 (77.8)			
Yes	40 (22.2)			
Type of infertility, *n* (%)				
Primary	134 (74.4)			
Secondary	46 (25.6)			

*SD* Standard Deviation

### Quality of life and depression in male and female dyads

As presented in Table 2, females’ depression was higher than their husbands (t(179) = 3.61, *P* < 0.001). On average, the mean total FertiQoL score of females was 5.5 lower than that of males (t(179) = 4.09, *P* < 0.001). In addition, regarding the subscales of the FertiQoL, females significantly scored lower than their husband on all domains of FertiQoL, except for the Social domain.

**Table 2:** Comparisons between male and female quality of life and depression scores (*n* = 180 couples)

	Male	Female	t_(179)_	*P*
Depression	4.82 ± 5.47	6.76 ± 5.78	3.61	< 0.001
Total FertiQoL Score	72.89 ± 15.94	67.36 ± 16.11	4.09	< 0.001
Emotional	67.34 ± 22.19	56.16 ± 22.33	5.68	< 0.001
Mind/Body	74.07 ± 20.00	67.31 ± 19.74	3.97	< 0.001
Relational	80.12 ± 16.51	77.08 ± 17.33	2.18	0.031
Social	70.05 ± 17.10	68.89 ± 18.74	0.71	0.476

*FertiQoL* Fertility Quality of Life

As shown in Table 3, males’ depression was correlated with both their own total FertiQoL score (*r* = − 0.608, *P* < 0.001) and female’ total FertiQoL score (*r* = − 0.232, *P* = 0.002). Females’ depression was also correlated with both their own total FertiQoL score (*r* = − 0.611, *P* < 0.001) and males’ total FertiQoL score (*r* = − 0.212, *P* = 0.004).

**Table 3:** Correlations coefficients among depression and quality of life in male and female dyads (*n* = 180 couples)

	1	2	3	4	5	6	7	8	9	10	11	12
Male												
1. Depression	1											
2. Quality of life	−0.61***	1										
3. Emotional	−0.53***	0.91***	1									
4. Mind/Body	− 0.59***	0.90***	0.82***	1								
5. Relational	−0.34***	0.69***	0.47***	0.46***	1							
6. Social	−0.55***	0.83***	0.67***	0.67***	0.46***	1						
Female												
7. Depression	0.18*	−0.21**	−0.17*	− 0.22**	− 0.15*	−0.17*	1					
8. Quality of life	−0.23**	0.36***	0.36***	0.32***	0.23**	0.28***	−0.61***	1				
9. Emotional	−0.12	0.25***	0.30***	0.23**	0.08	0.19*	−0.58***	0.89***	1			
10. Mind/Body	−0.22**	0.34***	0.33***	0.34***	0.18*	0.27***	−0.61***	0.88***	0.80***	1		
11. Relational	−0.22**	0.31***	0.27***	0.20**	0.39***	0.19**	−0.25**	0.63***	0.36***	0.37***	1	
12. Social	−0.21**	0.30***	0.30***	0.27***	0.15	0.27***	−0.54***	0.86***	0.70***	0.69***	0.44***	1

*p < 0.01; **p < 0.01; ***p < 0.001

### Impact of depression on quality of life at the dyadic level

The APIM results showed that the male’s depression as well as female’s depression exerted an actor effect on their own total QoL score (β = − 0.589, *p* < 0.001; β = − 0.588, *p* < 0.001, respectively). The same results were also found for all subscales of FertiQoL (Table 4).

**Table 4:** Actor and partner effects of depression on quality of life in infertile couples (*n* = 180 couples)

	Male			Female		
	β (SE)	t	P	β (SE)	t	P
Total FertiQoL score						
Actor’s depression	−0.589 (0.049)	12.06	< 0.001	−0.588 (0.049)	12.10	< 0.001
Partner’s depression	−0.108 (0.059)	1.81	0.070	−0.128 (0.059)	2.17	0.030
Emotional						
Actor’s depression	−0.521 (0.055)	9.51	< 0.001	−0.573 (0.051)	11.21	< 0.001
Partner’s depression	−0.075 (0.064)	1.18	0.238	−0.023 (0.062)	0.37	0.713
Mind/Body						
Actor’s depression	−0.570 (0.050)	11.31	< 0.001	−0.588 (0.049)	12.07	< 0.001
Partner’s depression	−0.115 (0.060)	1.91	0.057	−0.118 (0.059)	1.99	0.047
Relational						
Actor’s depression	−0.324 (0.067)	4.82	< 0.001	−0.218 (0.070)	3.10	0.002
Partner’s depression	−0.097 (0.071)	1.38	0.168	−0.182 (0.071)	2.57	0.010
Social						
Actor’s depression	−0.538 (0.053)	10.06	< 0.001	−0.518 (0.055)	9.49	< 0.001
Partner’s depression	−0.076 (0.063)	1.21	0.227	−0.120 (0.063)	1.90	0.057

*SE* Standard Error, *FertiQoL* Fertility Quality of Life

With regard to partner effects, however, only the male’s depression has a significant partner effect on female’s QoL (β = − 0.128, *p* = 0.030). Although the partner effect of female’s depression on male’s QoL was not statistically significant (β = − 0.108, *P* = 0.070), males whose wives had higher depression were more likely to indicate their own QoL was poorer. Regarding the subscales of the FertiQoL, only the partner effects of male’s depression on Mind/Body and Relational subscale scores were statistically significant (β = − 0.118, *p* = 0.047; β = − 0.182, *p* = 0.010, respectively) (Table 4).

The equality constraint tests were done to compare actor effects as well as partner effects between males and females, through the examination of the chi-square difference test. Constraining the actor effects to be equal did not significantly worsen the model fit (χ^2^(1) = 0.09, *P* = 0.759), indicating that the actor effects of depression on QoL were similar for males and females. The same results were also obtained for the partner effects (χ^2^(1) = 0.10, *P* = 0.746). We also tested the difference between actor effect and partner effect separately for male and female. For male participants, constraining the actor effect and partner effect to be equal does significantly worsen the model fit (χ2(1) = 27.60, *P* < 0.001), indicating that the actor effect of depression on QoL is larger than the partner effect of depression on QoL. The same results were also obtained for the female participants (χ^2^(1) = 22.09, *P* < 0.001).

## Discussion

To the best of our knowledge, this is the first study to apply the APIM approach to evaluate the impact of actor and partner depression on QoL in couples experiencing infertility. Although the majority of studies examining psychological distress and QoL both in infertile and fertile couples have focused on the impact of actor depression on QoL, there are growing calls to examine the partner effect of these variables.

As anticipated, females’ depression was higher than their husbands, suggesting that females tend to be more affected by infertility problem than males. This finding is in accordance with the results of previous studies [18, 19]. However, in two studies conducted in Iran [20] and Turkey [21], depression was unrelated to sex. In keeping with previous studies [22–27], females scored lower than their husbands on QoL. In other words, females’ QoL may be more considerably influenced by infertility problem than their husbands.

Based on correlational analysis, high correlation coefficients between males and females’ scores were observed. These confirm that male and female’s scores were adequately related to be deemed statistically non-independent, and so APIM approach would be more appropriate than conventional statistics.

Consistent with previous studies [5–7], the present study showed the considerable actor effect of depression on QoL. In other words, the greater level of depression that is experienced by either males or females with infertility contributes to poorer QoL for each individual.

The key result of the present study was the link between an individual’s depression and his/her spouse’s QoL. In accordance with our expectation, we observed that male’s depression negatively impacted female’s QoL. Contrary to our expectation, our study does not demonstrate a strong partner effect of females’ depression on their husbands’ QoL, although this effect was marginally significant, with *P* = 0.070.

As mentioned, these results again indicate that infertility and its treatment is shared problem, and so assessing couples from a system perspective can improve the knowledge of psychosocial complexity of infertility and enable health care professionals to develop interventions that help infertile couple manage psychological and social barriers to infertility and its treatment.

Our findings show that both actor and partner effects of depression on QoL were similar for males and females. Although the levels of depression differed between males and females, the associations between depression and QoL were not substantially different between males and females. This finding may indicate that both members of infertile couples share a similar mechanism through which depression influences QoL.

The current study has several limitations that should be mentioned. First, the generalization of the results may be affected by the relatively small sample size and single-center study design. Second, these results were found in a sample of Iranian infertile couples, and therefore may not generalize to other populations with different cultural experience. Third, another limitation of this study is the presence of multicollinearity. The PHQ-9 and FertiQoL tools we have used are significantly correlated, so in some way we are measuring very similar psychological adjustment constructs. Fourth, interactions that might exist between variables were not included in the models. Fifth, because of the cross-sectional nature of the study design, causal inferences between QoL and depression cannot be made. Sixth, this study is based on self-reported data that may be prone to social desirability bias.

## Conclusion

In spite of these limitations, the present study has yielded valuable information regarding the actors and partner effects of depression on QoL in male-female dyads experiencing infertility problems. The findings document that both actor and partner effects of depression on QoL are present in infertile couples, supporting the idea that a person’s depression can impact not only their own QoL but also his/her partner’s QoL. Moreover, interventions to reduce depression and to improve QoL should include both males and females. More complex studies in diverse populations and settings, particularly integrating mediation and/or moderation effects, are required to understand the relationship between depression and QoL.

## Abbreviations

APIM: Actor–Partner Interdependence Model
FertiQoL: Fertility Quality of Life
PHQ-9: Patient Health Questionnaire-9
QoL: Quality of Life
SEM: Structural Equation Modeling
